# Refinement of Magnetite Nanoparticles by Coating with Organic Stabilizers

**DOI:** 10.3390/nano6120228

**Published:** 2016-11-29

**Authors:** Monica Cîrcu, Alexandrina Nan, Gheorghe Borodi, Jürgen Liebscher, Rodica Turcu

**Affiliations:** 1 National Institute of Research and Development for Isotopic and Molecular Technologies, Donat 67-103, RO-400293 Cluj-Napoca, Romania; moni3_circu@yahoo.com (M.C.); borodi@itim-cj.ro (G.B.); liebscher@chemie.hu-berlin.de (J.L.); 2 Department of Chemistry, Humboldt-University Berlin, Brook-Taylor-Str. 2, 12489 Berlin, Germany

**Keywords:** magnetite nanoparticles, crystallite size, X-ray powder diffraction, coating, X-ray photoelectron spectroscopy, magnetic measurements

## Abstract

Magnetite nanoparticles are of great importance in nanotechnology and nanomedicine and have found manifold applications. Here, the effect of coating of magnetite nanoparticles with organic stabilizers, such as *O*-phosphoryl ethanolamine, glycerol phosphate, phospho-l-ascorbic acid, phospho-d,l-serine, glycolic acid, lactic acid, d,l-malic acid, and d,l-mandelic acid was studied. Remarkably, this procedure led to an improvement of saturation magnetization in three cases rather than to an unfavorable decrease as usually observed. Detailed X-ray powder diffraction investigations revealed that changes in the average crystallite occurred in the coating process. Surprisingly, changes of the average crystallite sizes in either direction were further observed, when the exposure time to the stabilizer was increased. These results imply a new mechanism for the well-known coating of magnetite nanoparticles with stabilizers. Instead of the hitherto accepted simple anchoring of the stabilizers to the magnetite nanoparticle surfaces, a more complex recrystallization mechanism is likely, wherein partial re-dispersion of magnetite moieties from the nanoparticles and re-deposition are involved. The results can help producers and users of magnetite nanoparticles to obtain optimal results in the production of core shell magnetite nanoparticles.

## 1. Introduction

Magnetic nanoparticles with tailored properties have found various important applications in nanomedicine, electronics, separation technology, catalysis, and in magnetic sealing. Amongst them, magnetite (Fe_3_O_4_) nanoparticles (MNPs) are most prominent [1,2,3,4,5,6]. They are easy to prepare at low costs and are much less toxic than other magnetic nanoparticles [7,8]. For all applications, they must meet crucial property requirements, such as colloidal and chemical stability and, in particular, a high magnetization. In most applications, MNPs have to be coated with stabilizers in order to improve colloidal and chemical stability and to allow further functionalization [5,8]. Coating can be done by metals (e.g., gold) or inorganic materials (e.g., silica), by organic polymers (e.g., polyacrylamides or esters, polyethylene amine, polyethylene glycol, polydopamine) or most often by monomers (e.g., fatty acids, citric acid, phosphonic acids, or phosphates). The coating can be performed in situ during the preparation of the MNPs (e.g., by oleylamine/oleic acid in the thermal decomposition method) [9] or post synthetically by mixing the MNP with the respective stabilizer (e.g., fatty acids in the co-precipitation method) [4]. Various studies revealed that coating with stabilizers can affect the size, the shape, and the magnetism of the MNP as can other factors such as temperature, concentration, type of anions, ionic strength, and pH or exposure to an external magnetic field [1,4,5,10,11,12,13,14]. It is generally accepted that coating by stabilizers usually causes a decrease in saturation magnetization measured in emu/g because of the increase in total mass. Information about the effect of coating on the crystallite structure found in the magnetite cores are still missing. There is one report about the influence of citrate on the formation of Fe-oxides revealing that citrate hampers the crystallization process [15]. In the present study, the effect of a selection of chelating stabilizers on the average crystallite size in MNP is investigated. The results provide information about the suitability of various chelating stabilizers. Three cases of magnetite core shell structures were found with a significantly increased saturation magnetization. The results further allow understanding the process of surface coating of MNP in a hitherto unknown way.

## 2. Results and Discussion

**Figure 1:**
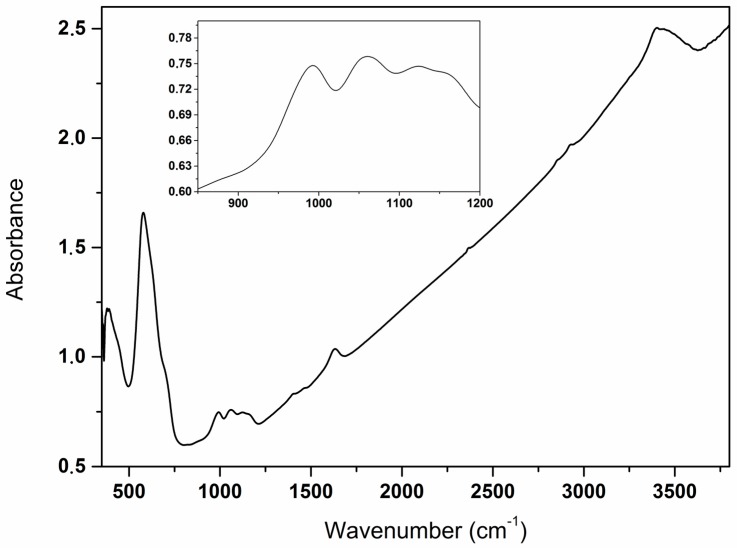
Fourier transform infrared spectroscopy (FTIR) spectrum of **3b** (Fe_3_O_4_ covered with glycerol phosphate); inset shows an amplified region between 800 and 1200 cm^−1^.

**Scheme 1:**
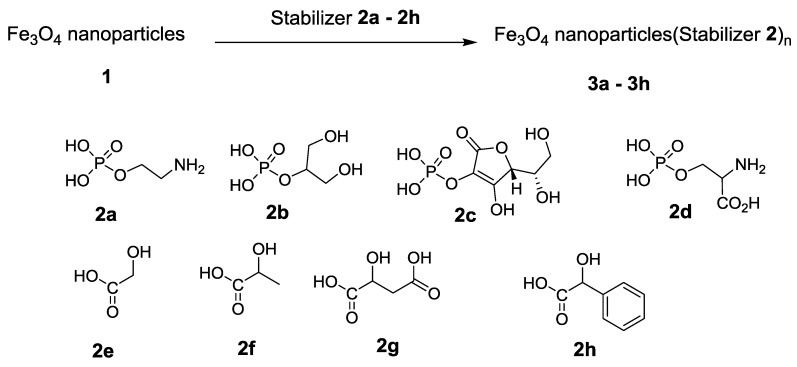
Coating of magnetic nanoparticles **1** with stabilizers **2** to magnetic naoparticles **3**.

The co-precipitation method using FeCl_2_ and FeCl_3_ in aqueous ammonia, which has been most often applied to prepare MNP, was also used in our study. This well-known method leads to MNP **1** in a short reaction time (usually less than 1 h) [1,16]. As a reference, samples of these ‘naked’ MNP **1** were collected after 1, 3 and 24 h. In order to investigate the effect of surface coating onto the naked MNP **1**, the following stabilizer were added after 1 h: *O*-phosphoryl ethanolamine **2a** [17], glycerol phosphate **2b** [18], phospho-l-ascorbic acid **2c** [18], phospho-d,l-serine **2d**, glycolic acid **2e** [19,20], d,l-lactic acid **2f**, malic acid **2g**, and d,l-mandelic acid **2h** (Scheme 1). The resulting MNP **3** were separated by magnetic decantation after 3 or 24 h. Most of these types of coatings of MNP (except **3f**, **3g**, **3h**) were reported before and characterized to some extent. The products **3** obtained here were analyzed by X-ray powder diffraction (XRPD), vibrating sample magnetometry (VSM), Fourier transform infrared spectroscopy (FTIR), and in selected cases by X-ray photoelectron spectroscopy (XPS), thermogravimetric analysis (TGA), and transmission electron microscopy (TEM). FTIR spectra of all samples (see Supplementary Material Figures S1–S7 and Figure 1) confirmed the presence of magnetite (intensive bands at 580 cm^−1^) and organic material (typical bands at 1050 cm^−1^ specific for P–O bond and around 1650 cm^−1^ attributed to the C=O in **3c**–**3h**, respectively). Analysis of the IR-region 900–1200 cm^−1^ typical for phosphate suggested that bidentate POR(OFe)_2_ motifs are found at the surface of MNP **3a**, **3b**, **3c** and **3d** (three bands at around 922–998 cm^−1^, 1017–1030 cm^−1^, 1089–1180 cm^−1^, see also inset in Figure 1). However, there are also additional bands of other motifs found in this region [21,22,23].

**Figure 2:**
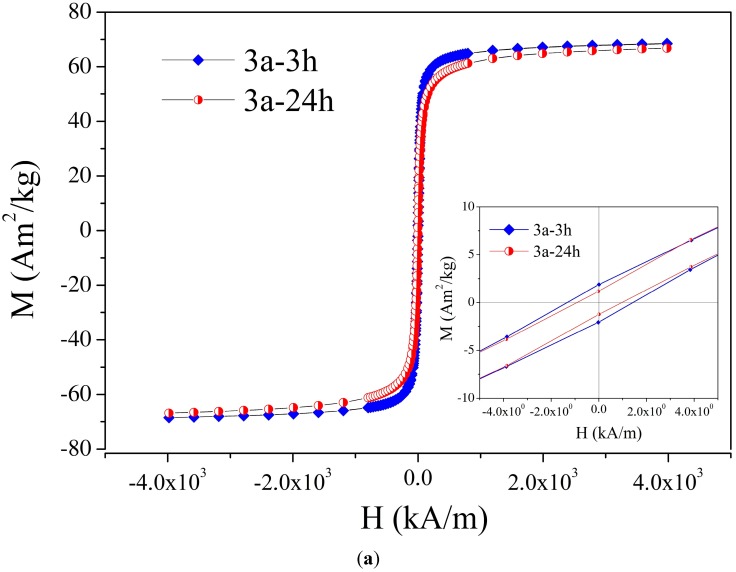

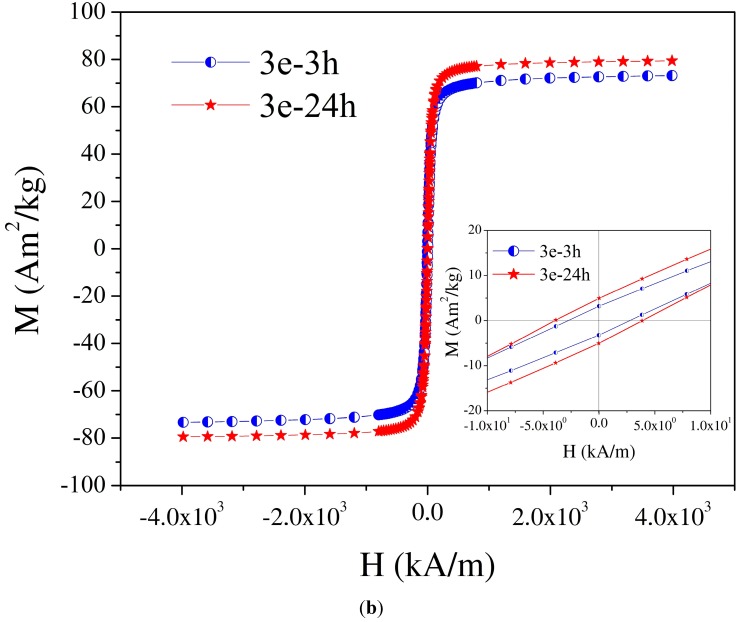
Magnetization versus applied magnetic field of magnetic nanoparticles (MNPs) at room temperature: (**a**) **3a**-3 h, **3a**-24 h; (**b**) **3e**-3 h, **3e**-24 h.

Saturation magnetization values of the magnetic nanoparticles **3** coated with various stabilizers are in the range 59.8–80 emu/g (see Table 1). The magnetization curves show small coercivity values of 0.5–3.9 kA/m depending on the crystallite sizes. Coercivity is very sensitive to the magnetic nanoparticles’ size. In this context, the difference between the two characteristic sizes has to be mentioned: single-domain size *D_sd_* and critical size for superparamagnetic behavior *D*_spm_ [24,25]. For small volumes (sizes), the magnetic reversal energy is small enough that the magnetic moment becomes unstable or thermally activated, which corresponds to superparamagnetic behavior. For a typical quasistatic measuring regime at room temperature, the characteristic size for superparamagnetism results to be significantly (approximately two to four times) less than the single domain size for iron oxide nanoparticles (see picture 3 [24] and Table 1.3 in [25]). Indeed, data reported in the literature show that a small coercivity could appear for single-domain magnetic nanoparticles with sizes *D* in the range: *D*_spm_ < *D* < *D*_sd_ [24,26], the full magnetization curve shows small coercivity. Thus, although the MNP **3** have sizes corresponding to single-domain magnetite nanoparticles (see TEM investigations below), the magnetization shows small coercivity. Magnetization curves with insets of coercivity of MNPs **3a**-3 h, **3a**-24 h and **3e**-3 h, **3e**-24 h are found in Figure 2a,b, respectively. The saturation magnetization values of MNP **3e** (74 emu/g), **3g** (71.9 emu/g) and **3h** (74.6 emu/g) covered with glycolic acid, malic acid, or mandelic acid, respectively, are even higher than those of the starting MNP **1**. This fact is of great importance for practical application of MNP, where a high magnetization is pursued. Since coating of MNP with organic stabilizers causes a decrease in saturation magnetization due to the increase in total mass (*v. i.*), the observed increase must be caused by changes in the magnetite core during the transformation from **1** into **3**. All of these ligands **2e**, **2g**, **2h**, causing an increase in saturation magnetization contain a α-hydroxy acid motif, which is able to form chelate complexes with the magnetite. At present, it is unclear why d,l-lactic acid (MNP **3f**: 46.4 emu/g) did not result in such an increase in saturation magnetization.

**Figure 3:**
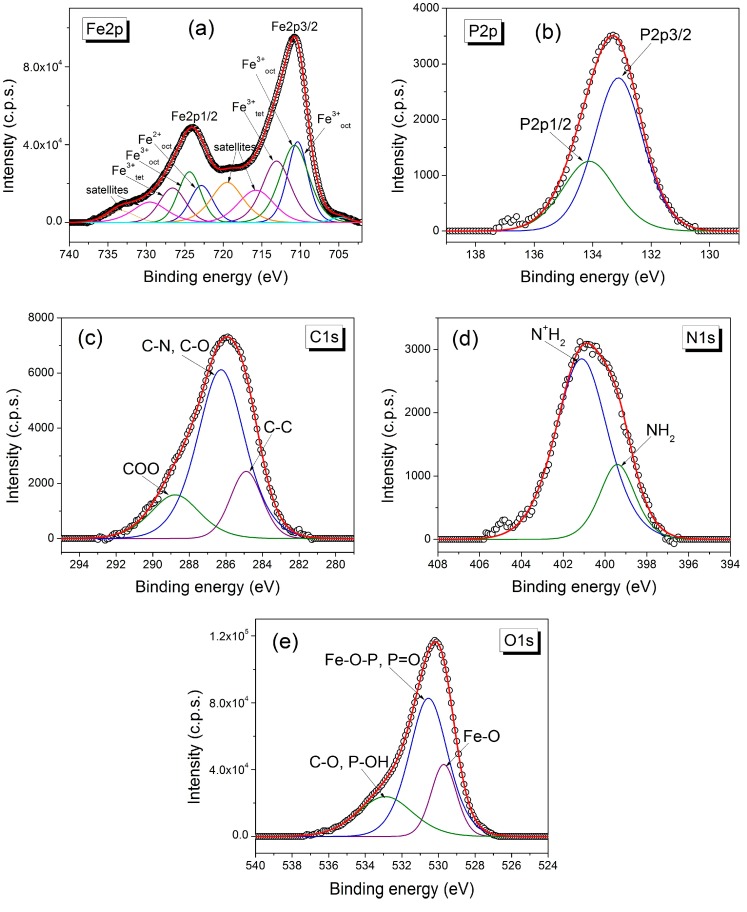
High resolution X-ray photoelectron spectroscopy XPS spectra of (**a**) Fe2p, (**b**) P2p, (**c**) C1s, (**d**) N1s, (**e**) O1s core-levels of magnetic nanoparticles **3a**-3 h.

XPS analysis was used to confirm the coating of MNPs with stabilizers (Figure 3 and Figures S8–S11 from Supplementary Materials). As an example, XPS spectra of Fe2p, O1s, P2p, C1s, N1s core-levels of MNP **3a**-3 h are shown in Figure 3. The Fe2p spectrum contains the doublet Fe2p3/2 (710.7 eV) and Fe2p1/2 (724.1 eV) and their satellites. Its best fit contains contributions from Fe^2+^ octahedrally and Fe^3+^ ions either octahedrally or tetrahedrally coordinated, characteristic for the inverse spinel structure of magnetite [27]. The coating of MNP **3a**-3 h with *O*-phosphoryl ethanolamine is confirmed by the P2p, O1s and N1s XPS spectrum. In the first case, a doublet of P2p3/2 and P2p1/2 is found. The best fit for O1s spectrum was obtained with three components corresponding to oxygen atoms from (i) Fe–O (529.7 eV); (ii) P=O, Fe–O–P (530.5 eV); (iii) C–O, P–OH (532.9 eV). The C1s spectrum contains an intense components ascribed to C–N, C–O groups (286.3 eV) of *O*-phosphoryl ethanolamine and also components located at 284.9 eV and 288.8 eV, which could be due to a contamination layer. The N1s spectrum was deconvoluted into two peaks located at 399.4 eV and 401.1 eV corresponding to NH_2_ and N^+^H_2_, respectively.

The atomic concentration ratio Fe^3+^/Fe^2+^ calculated from XPS peak areas is 1.7 for MNP **3a**-3 h and **3f**-3 h, 2.2 for **3g**-3 h, 2.3 for **3h**-3 h, while all other MNP **3** (not shown) exhibit ratios of 2, as expected for magnetite. XPS analysis of MNP **3d** (Figure S8) showed that part of the phosphor-d,l-serine was deaminated under the influence of the magnetite, presumably resulting in the respective α-keto acid. Such a type of oxidative deamination under the influence of MNP was observed before with serine [28].

**Figure 4:**
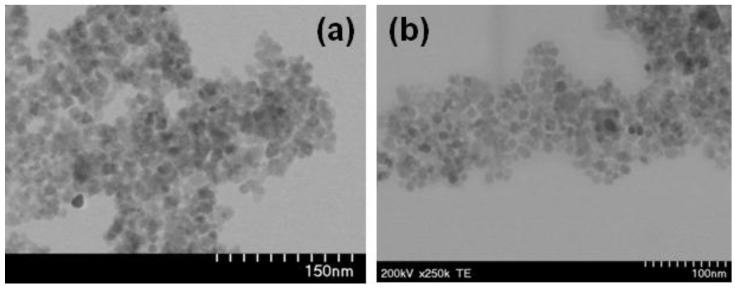
Transmission electron microscopy (TEM) images of magnetic nanoparticles **3a**-3 h (**a**) and MNP **2h**-3 h (**b**).

Shape and size of selected MNP were investigated by TEM (Figure 4, Figures S12 and S13). It turned out that the nanoparticle size is well below 50 nm, corresponding to single-domain magnetite nanoparticles. For example, diameter ranges of 19–22 nm and 11–14 nm were found for MNP **3a**-3 h and MNP **3h**-3 h, respectively. Their shape is nearly spherical. Partial aggregation is probably caused by the preparation of the samples for TEM measurement.

**Figure 5:**
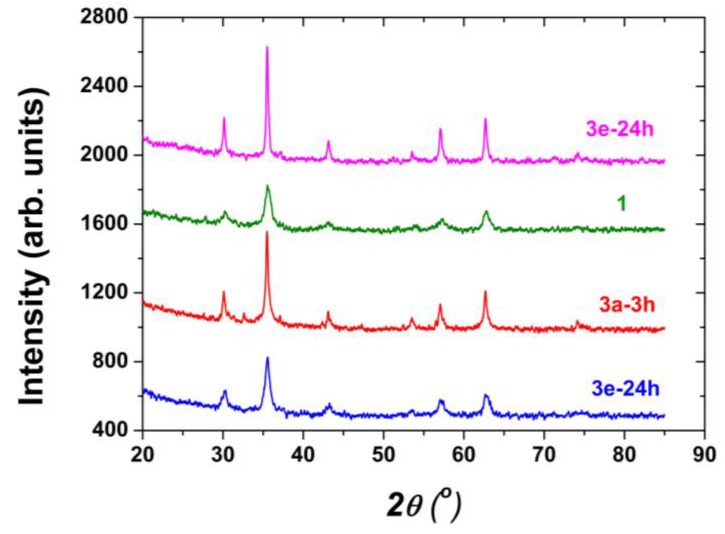
X-ray powder diffraction (XRPD) patterns of magnetic nanoparticles **3a**-24 h, **3a**-3 h, **1** and **3e**-24 h.

XRPD of all MNP was performed, some results are shown in Figure 5. It can be noted that reflection peaks of samples **3a**-24 h and **1** are broad (small crystallite sizes) with small intensities, whereas others like the diffraction peaks of **3a**-3 h and **3e**-24 h are narrow, having higher intensities revealing bigger crystallite size and better formed crystalline lattices.

Considering all the results obtained in XRPD applying Scherrer equation shown in Table 1, it appeared that, in comparison to MNP **1,** the average crystallite size of MNP **3** observed after 3 h exposure time of MNP **1** to stabilizers **2a**-h increased. It can vary considerably from 32 nm (**3e**, entry 11) to 15.2 nm (**3h**, entry 16) as compared with 11.5 nm (entry 1) for non-stabilized MNP **1**. The following order was observed **3e**, **3g** > **3a**, **3b**, **3d**, **3f** > **3c**, **3h** > **1**. Except for MNP **3h**-3 h (Table 1, entry 17), the strength of the saturation magnetization of MNP **3** obeyed a similar sequence but, altogether the values did not differ so much ranging from 74.0 emu/g for **3e** (entry 11) to 64.6 emu/g for **3d** (entry 9). However, the very high value of 74.6 emu/g found for MNP **3h**-3 h (Table 1, entry 17) is surprising in consideration of its small average crystallite size (15.2 nm, Table 1, entry 17) of MNP **3**. In addition to the Scherrer equation, the Williamson-Hall plot [29,30] was applied to obtain crystallize sizes and strain of the MNP **3** (Table S1). These values are generally a bit smaller but show the same trends as the Scherrer results, thus manifesting our conclusions drawn from Table 1. Remarkable changes in strain with exposure time were only observed with MNP **3c** and **3h**, i.e., examples without drastic changes in the crystallite size and thus not of particular importance in the present context.

Strikingly, the average crystallite size of MNP **3** changed in some cases when the exposure time of the MNP **1** to the stabilizers **2a**–**f** was increased from 3 h to 24 h (Table 1). While MNP **3b**, **3g** and **3h** did not show a significant change, the other examples either had decreased crystallite sizes (**3a**: 25.7 nm → 13.6 nm; **3c**: 17.1 nm → 13.3 nm; **3f**: 25 nm → 20 nm) or increased (**3e**: 32 nm → 39.4 nm; **3d**: 24.9 nm → 27.2 nm). A uniform relation between the trends found in the change of crystallite size as compared with the saturation magnetization when changing the exposure times from 3 to 24 h was observed, except for **1** and **3f**. The small decrease of saturation magnetization from 69.6 emu/g to 67.8 emu/g for the phosphoryl ethanolamine coated MNP **3a**-3 h and MNP **3a**-24 h is somewhat unexpected as the average crystallite size dropped considerably from 25.7 nm to 13.6 nm (*v. i.*).

TGA was performed from samples **3a**-3 h, **3a**-24 h, **3e**-3 h, **3e**-24 h, **3f**-3 h, **3g**-3 h and **3h**-3 h revealing the composition of the MNP with respect to magnetite core, organic shell and traces of adsorbed water. The magnetite content was used to normalize the measured magnetization values of these samples (see footnotes Table 1). Naturally, the normalized values are somewhat higher, but follow the same trends as found with the measured values. Remarkably, the percentage of organic shell can vary considerably depending on the type of stabilizer from 1.4% (**3e**-24 h) to 25% (**3f**-3 h). It is further worth mentioning that the amount of the shell decreased with increasing exposure time in cases of compound **3a**-3 h (8.4%) to **3a**-24 h (4.8%) and **3e**-3 h (2.9%) to **3e**-24 h (1.4%).

**Scheme 2:**
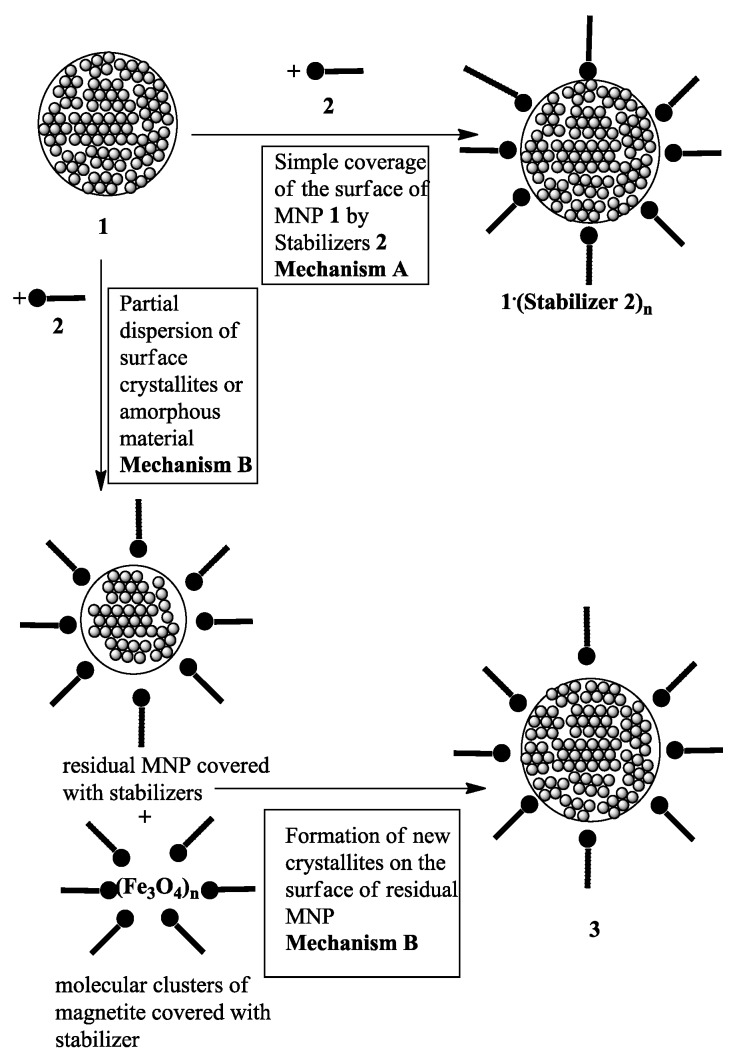
Mechanisms of coating magnetic nanoparticles **1** with stabilizers **2**.

The unusual results observed in the effect of stabilizers on the crystallite size demand revisiting the hitherto accepted mechanism of stabilization of magnetite nanoparticles. So far, it is assumed that such MNP react by their surface OH groups with the stabilizing ligand forming salt-like or chelate like structures with the ligand resulting in core shell nanostructures (Scheme 2, Mechanism A). This mode of interaction, however, does not affect the crystallite site and thus cannot explain our results. If the average crystallite size in the MNP of **1** changes during interacting with a stabilizer **2,** there must be at least a partial break down of the original crystallites found in the starting MNP **1**. It is known that MNPs are not homogeneous, consisting not only of spinel crystallites, but they can also contain crystal defects and amorphous material [2]. Therefore, molecular clusters of magnetite could be removed from the surface of the original MNP **1** by complex formation with the stabilizing ligand **2** and could later on re-deposit to form new crystallites (Scheme 2, Mechanism B), which can differ in size and properties from those in the starting MNP **1**. Since the newly formed crystallite can either be larger or smaller, the average crystallite size of the MNP **3** changes accordingly. In this way, the observed changes in average crystallite sizes with increasing exposure times (see Table 1) are understandable. It is well known that magnetite nanostructures can change in aqueous medium without the interaction with stabilizers by re-dissolving Fe(II) ions or by oxidation [3,31]. However, under the oxygen-free conditions used by us (argon as inter atmosphere, see experimental procedure) oxidation is unlikely. This is verified by only small changes in crystallite sizes of naked MNP **1** with time (Table 1, entries 1–2). Thus, the changes observed in the presence of certain stabilizers under argon atmosphere (Table 1, entries 3–4, 11–12, 13–14) are unlikely to be caused by oxidation.

Arguments that the changes in the average crystallite sizes are caused just by irreversible removal of magnetically non-separable small or defect crystallites or molecular clusters from the starting MNP **1** by the stabilizer **2** are not in agreement with our results. Since smaller units are more easily dispersed from the surface of the MNP **1** and thus should cause an increase of average crystallite size, the observed decrease (Table 1, entries 3/4 and 7/8, 13/14) is in contradiction to this argument. The observed decrease in average crystallite size during the transformation of MNP **1** into MNP **3** also rules out the argument that smaller particles of **1**, after coating with the stabilizer, were not collected in the magnetic separation due to lowered saturation magnetization caused by the increase in weight. Thus, there is sound evidence for our proposed alternative mechanism B (Scheme 2). Possibly, mechanism B is also responsible for the observed decrease in percentage of organic layers with increasing exposure time as determined by TGA (see Table 1. entries 3/4, footnotes a, d and 11/12, footnotes f, h).

## 3. Materials and Methods

All chemicals used in the experiments were purchased from Sigma-Aldrich, Munich, Germany and Acros, Geel, Belgium. FTIR spectra were measured on a JASCO FTIR 6100 spectrophotometer, Tokyo, Japan. The magnetic measurements were performed at room temperature with a sweep rate of the magnetic field 0.05 T/min by using a Vibrating Sample Magnetometer Cryogenics, London, UK. The chemical surface analysis of the functionalized magnetic nanoparticles was performed by X-ray Photoelectron Spectroscopy (XPS). XPS spectra were recorded using a XPS spectrometer SPECS from Berlin, Germany equipped with a dual-anode X-ray source Al/Mg, a PHOIBOS 150 2D CCD hemispherical energy analyzer and a multi-channeltron detector with vacuum maintained at 1.9 × 10^−9^ torr. The Al Kα X-ray source (1486.6 eV) operated at 200 W was used for XPS investigations. The XPS survey spectra were recorded at 30 eV pass energy, 0.5 eV/step. The high-resolution spectra of the individual elements were recorded by accumulating 10–15 scans at 30 eV pass energy and 0.1 eV/step. The powdered sample was pressed on an indium foil to allow the XPS measurements. A cleaning of the samples surface was performed by argon ion bombardment (300 V). Data analysis and curve fitting were performed using Casa XPS software with a Gaussian–Lorentzian product function and a non-linear Shirley background subtraction from Berlin, Germany. X-ray Power Diffraction (XRPD) measurements were performed with a Bruker D8 Advance X-ray diffractometer, with a Ge (111) monochromator for Cu-Kα1 radiation (λ = 1.5406 Å) having the source power of 40 kV and 40 mA, at room temperature and with a LynxEye position sensitive detector, from Karlsruhe, Germany. Crystallite sizes shown in Table 1 were determined using Scherrer relationship [16]: *D* = 0.9 [32]. As an alternative, the evolution of crystallite size and strain was carried out using the Williamson-Hall plot (results in Table S1). TEM images were recorded on a HD-2700 STEM, Tokyo, Japan at 80-200 kV field emisison gun 0.204 nm guaranteed (at a magnification of ×4,000,000). TGA analyses were performed with a Mettler Toledo 851E instrument, from Greifensee, Switzerland applying STARe Excellence Software. Nitrogen was used as inert gas (50 mL/min). The heating rate was 10 °C/min from 25 °C to 800 °C.

MNP **1** were synthesized by the well-known co-precipitation method from FeCl_2_ and FeCl_3_ in the presence of aqueous ammonia as follows: In a three-neck round bottom flask provided with a condenser, a dropping funnel and a thermometer, concentrated aqueous ammonia (200 mL) were placed and warmed up to 80 °C under argon. A solution of FeCl_2_ (5.94 g, 0.03 mol) and FeCl_3_ (16.2 g, 0.06 mol) in distilled water (80 mL) was added drop wise at 80 °C. After stirring the reaction mixture at 80 °C with a magnetic stirrer for an hour, the reaction mixture was allowed to cool down to room temperature. The stabilizer **2** (0.15 mol) was added and the mixture was stirred at room temperature under argon atmosphere for 3 h or 24 h (see Table 1). The MNP **3** formed were separated with the aid of an external NdFeB magnet and washed rigorously first with water and finally with acetone. They were allowed to dry at room temperature.

## 4. Conclusions

Magnetite nanoparticles **1** were synthesized by the widely used co-precipitation method from Fe(II) and Fe(III) and later on coated with *O*-phosphoryl ethanolamine, glycerol phosphate, phospho-l-ascorbic acid, phospho-d,l-serine, glycolic acid, lactic acid, malic acid, or mandelic acid as stabilizers. MNP **3** of significantly increased saturation magnetization were obtained with glycolic acid, malic acid, or mandelic acid. Thus, these systems are of particular interest for practical application. Detailed XRPD investigations revealed that the coating of **1** gave rise to an increase in the average crystallite size. Furthermore, unexpected changes of the average crystallite sizes with changing exposure times were observed, giving evidence for a new mechanism of coating of magnetic nanoparticles with stabilizers. Instead of the hitherto accepted simple anchoring of the stabilizers to the magnetite nanoparticle surfaces, a more complex recrystallization mechanism is likely to occur including re-dispersing magnetite moieties from the nanoparticles and re-deposition. The results lead to important insights in the mode of interaction of stabilizers with magnetite nanoparticles and provide important information to the manufacturing and application of magnetic nanoparticles in different areas. Currently, we investigate the effect of chirality of stabilizers on the properties of magnetite crystallites.

## Figures and Tables

**Table 1:** Average crystallite sizes calculated with Scherrer equation and saturation magnetization of magnetic nanoparticles **1** and **3**.

Entry	Magnetic Nanoparticles	Stabilizer/Reaction Time	Description	X-ray Powder Diffraction (XRPD) (Average Crystallite Size)	Saturation Magnetizations Values (emu/g)
1	**1**-3 h	**-**/3 h	Naked Fe_3_O_4,_ 3 h	11.5 nm	68.5
2	**1**-24 h	**-**/24 h	Naked Fe_3_O_4_, 24 h	14.1 nm	65.7
3	**3a**-3 h ^a^	**2a**/3 h	Fe_3_O_4_ covered with *O*-phosphoryl ethanolamine, 3 h	25.7 nm ^b^	69.6 ^b,c^
4	**3a**-24 h ^d^	**2a**/24 h	Fe_3_O_4_ covered with *O*-phosphoryl ethanolamine, 24 h	13.6 nm ^b^	67.8 ^a,e^
5	**3b**-3 h	**2b**/3 h	Fe_3_O_4_ covered with glycerol phosphate, 3 h	25 nm	68.8
6	**3b**-24 h	**2b**/24 h	Fe_3_O_4_ covered with glycerol phosphate, 24 h	27.9 nm	70.8
7	**3c**-3 h	**2c**/3 h	Fe_3_O_4_ covered with phospho-l-ascorbic acid, 3 h	17.1 nm	68.2
8	**3c**-24 h	**2c**/24 hr	Fe_3_O_4_ covered with phospho-l-ascorbic acid, 24 h	13.3 nm	59.8
9	**3d**-3 h	**2d**/3 h	Fe_3_O_4_ covered with phospho-d,l-serine, 3 h	24.6 nm ^b^	64.6
10	**3d**-24 h	**2d**/24 h	Fe_3_O_4_ covered with phospho-d,l-serine, 24 h	27.2 nm	65.4
11	**3e**-3 h ^f^	**2e**/3 h	Fe_3_O_4_ covered with glycolic acid, 3 h	32.0 nm	74.0 ^g^
12	**3e**-24 h ^h^	**2e**/24 h	Fe_3_O_4_ covered with glycolic acid, 24 h	39.0 nm ^b^	80 ^e,i^
13	**3f**-3 h ^j^	**2f**/3 h	Fe_3_O_4_ covered with lactic acid, 3 h	25 nm	45.5 ^a,k^
14	**3f**-24 h	**2f**/24 h	Fe_3_O_4_ covered with lactic acid, 24 h	20 nm	47.0 ^a^
15	**3g**-3 h ^l^	**2g**/3 h	Fe_3_O_4_ covered with malic acid, 3 h	31.7 nm	71.9 ^m^
16	**3g**-24 h	**2g**/24 h	Fe_3_O_4_ covered with malic acid, 24 h	30.7 nm	68.1
17	**3h**-3 h ^n^	**2h**/3 h	Fe_3_O_4_ covered with mandelic acid, 3 h	15.2 nm	74.6 ^o^
18	**3h**-24 h	**2h**/24 h	Fe_3_O_4_ covered with mandelic acid, 24 h	15.3 nm	73

^a^ according to thermogravimetric analysis (TGA) 90.4% magnetite, 1.2% water, 8.4% organic shell; ^b^ average value of two samples; ^c^ 77.0 normalized to magnetite core; ^d^ according to TGA 94.3% magnetite, 0.8% water, 4.9% organic shell; ^e^ 71.9 normalized to magnetite core; ^f^ according to TGA 96.5% magnetite, 0.6% water, 2.9% organic shell; ^g^ 76.7 normalized to magnetic core; ^h^ according to TGA 98% magnetite, 0.6% water, 1.4% organic shell; ^i^ 81.6 normalized to magnetite core; ^j^ according to TGA 71.8% magnetite, 2.7% water, 25.5% organic shell; ^k^ 63.4 normalized to magnetite core; ^l^ 87.6% magnetite, 1.8% water, 10.6% organic shell; ^m^ 82.1 normalized to magnetite core; ^n^ according to TGA 89.1% magnetite, 0.6% water, 10.3% organic shell; ^o^ 83.7 normalized to magnetite core (89.1%).

## Supplementary Materials

The following material is available online at http://www.mdpi.com/2079-4991/6/12/228/s1.
