# Supplementary material for: Refinement of Magnetite Nanoparticles by Coating with Organic Stabilizers
Source: Nanomaterials (Basel). 2016 Nov 29;6(12):228. doi: 10.3390/nano6120228 (PMC5302720; doi:10.3390/nano6120228)
Supplement: Supplementary file 1 [file nanomaterials-06-00228-s001.pdf]

Supplementary Materials: Refinement of Magnetite Nanoparticles by Coating with Organic Stabilizers

Monica Cîrcu, Alexandrina Nan, Gheorghe Borodi, Jürgen Liebscher and Rodica Turcu

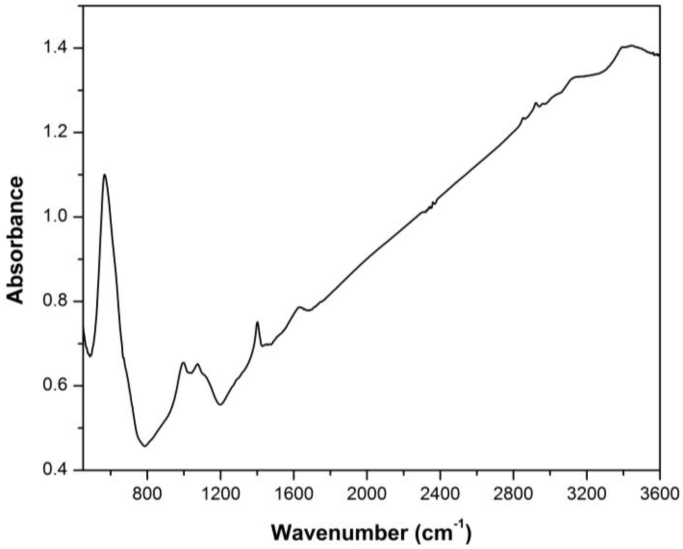

Figure S1. FTIR spectrum of **3a** (Fe_3O_4 covered with *O*-phosphoryl ethanolamine).

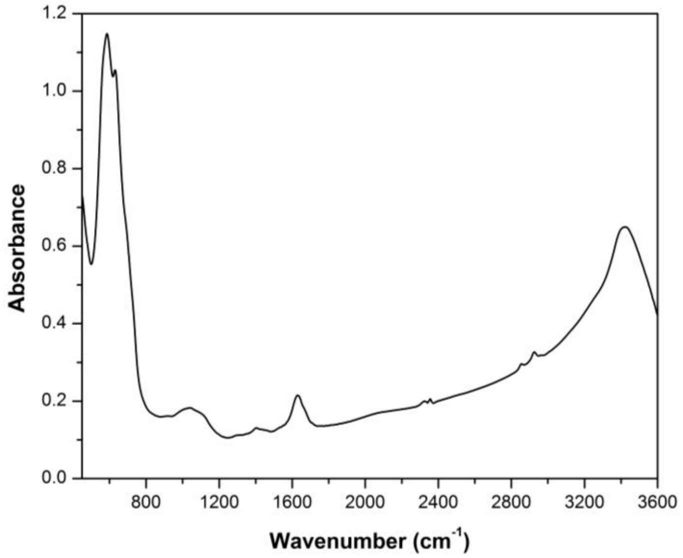

Figure S2. FTIR spectrum of **3c** (Fe_3O_4 covered with phospho-L-ascorbic acid).

Table S1. Average crystallite sizes and saturation magnetization of MNP 1 and 3 including crystallite sizes and strains obtained by applying Williamson-Hall method (right column).

Entry	MNP	Description	XRPD (Average Crystallite Size-Scherrer)	VSM (Magnetization)/emu/g	Crystallite Size and Strain $\beta_{tot}\cos\theta = C\varepsilon\sin\theta + K\lambda/L$	
					Crystallite Size	Strain (ε. 10^{-4})
1	**1**	Naked Fe_3O_4, 3 h	11.5 nm	68.5	11.0 nm	30
2	**1**	Naked Fe_3O_4, 24 h	14.1 nm	65.7	14.2 nm	9.5
3	**3a**-3 h	Fe_3O_4 covered with *O*-phosphoryl ethanolamine, 3 h	25.7 nm [a]	69.6 [a]	20.1 nm	15
4	**3a**-24 h	Fe_3O_4 covered with *O*-phosphoryl ethanolamine, 24 h	13.6 nm [a]	67.8 [a]	13.4 nm	20
5	**3b**-3 h	Fe_3O_4 covered with glycerol phosphate, 3 h	25 nm	68.8	20.1 nm	14
6	**3b**-24 h	Fe_3O_4 covered with glycerol phosphate, 24 h	27.9 nm	70.8	22.6 nm	16
7	**3c**-3 h	Fe_3O_4 covered with phospho-L-ascorbic acid, 3 h	17.1 nm	68.2	16.5 nm	10
8	**3c**-24 h	Fe_3O_4 covered with phospho-L-ascorbic acid, 24 h	13.3 nm	59.8	13.0 nm	3
9	**3d**-3 h	Fe_3O_4 covered with phospho-D,L-serine, 3 h	24.6 nm [a]	64.6	20.6 nm	12
10	**3d**-24 h	Fe_3O_4 covered with phospho-D,L-serine, 24 h	27.2 nm	65.4	27.0 nm	8
11	**3e**-3 h	Fe_3O_4 covered with glycolic acid, 3 h	32.0 nm	74.0	27.0 nm	15
112	**3e**-24 h	Fe_3O_4 covered with glycolic acid, 24 h	39.0 nm [a]	80	34.7 nm	10
13	**3f**-3 h	Fe_3O_4 covered with lactic acid, 3 h	25 nm	45.5 [a]	16.4 nm	17
14	**3f**-24 h	Fe_3O_4 covered with lactic acid, 24 h	20 nm	47.0 [a]	12.8 nm	23
15	**3g**-3 h	Fe_3O_4 covered with malic acid, 3 h	31.7 nm	71.9	23.6 nm	20
16	**3g**-24 h	Fe_3O_4 covered with malic acid, 24 h	30.7 nm	68.1	25.6 nm	19
17	**3h**-3 h	Fe_3O_4 covered with mandelic acid, 3 h	15.2 nm	74.6	14.6 nm	3
18	**3h**-24 h	Fe_3O_4 covered with mandelic acid, 24 h	15.3 nm	73	14.4 nm	15

[a] average value of two samples.

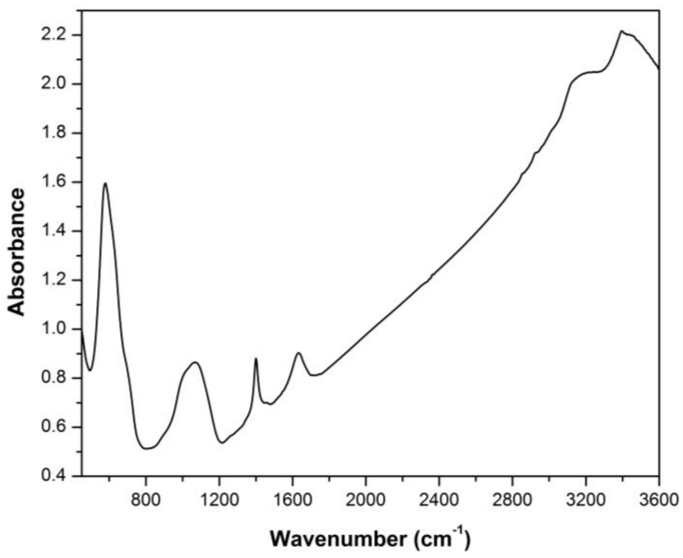

Figure S3. FTIR spectrum of **3d** (Fe_3O_4 covered with phospho-D,L-serine).

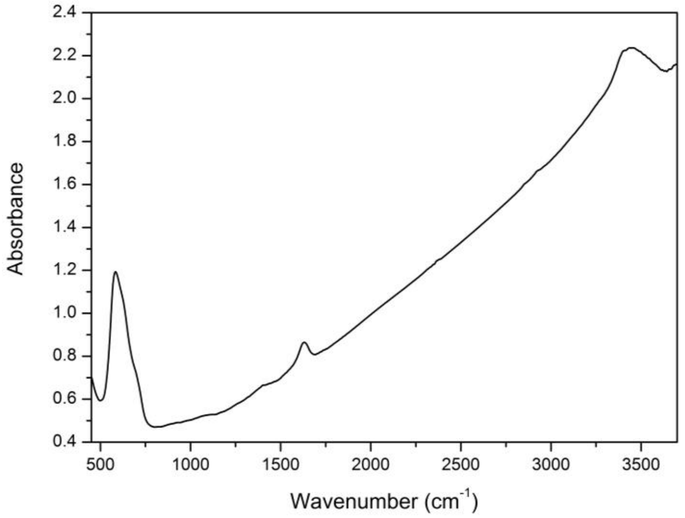

Figure S4. FTIR spectrum of **3e** (Fe_3O_4 covered with glycolic acid).

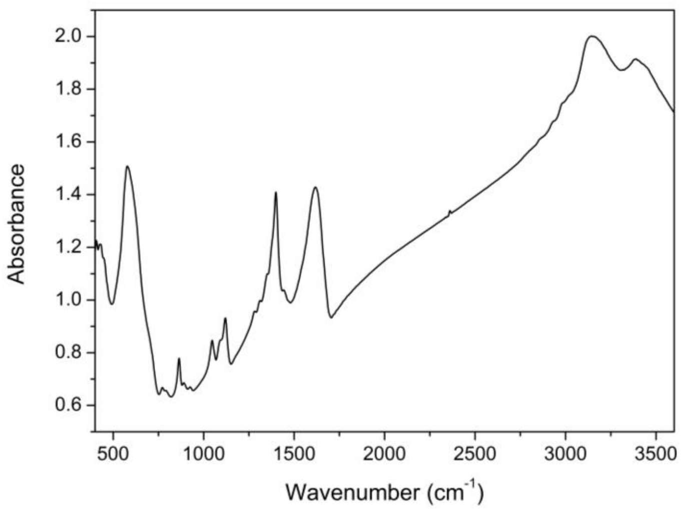

Figure S5. FTIR spectrum of **3f** (Fe_3O_4 covered with lactic acid).

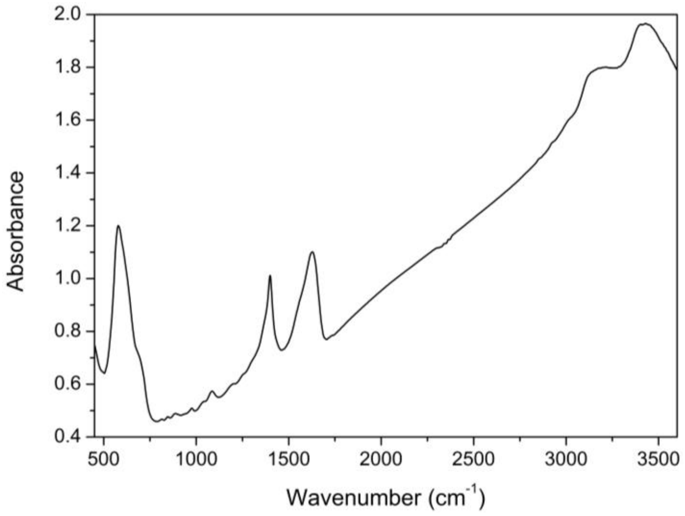

Figure S6. FTIR spectrum of **3g (**Fe_3O_4 covered with malic acid).

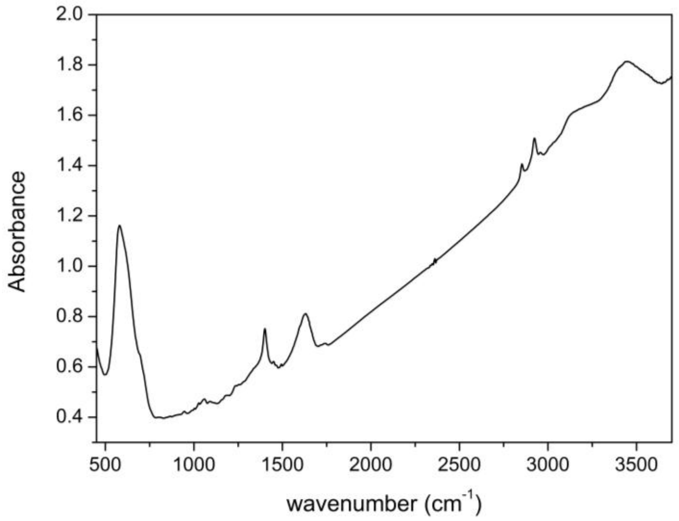

Figure S7. FTIR spectrum of **3h** (Fe_3O_4 covered with mandelic acid).

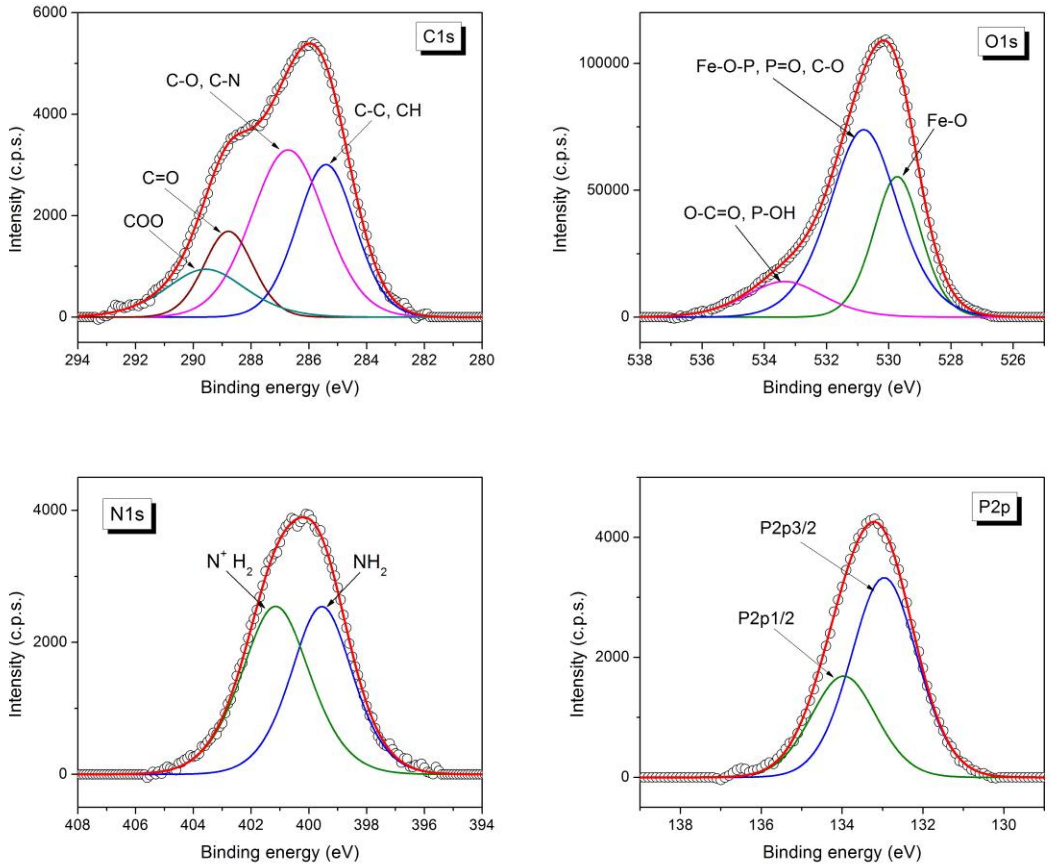

Figure S8. XPS spectra of **3d**-3 h.

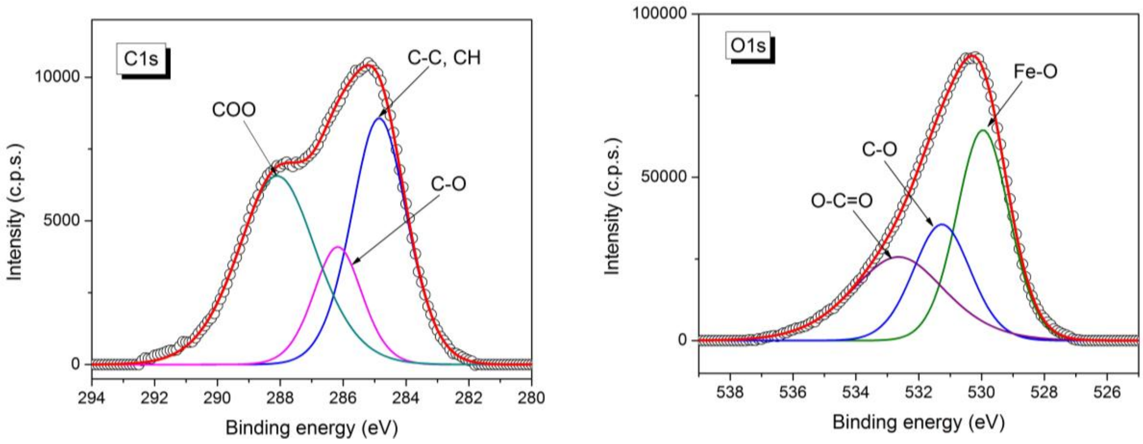

Figure S9. XPS spectra of **3f**-3 h.

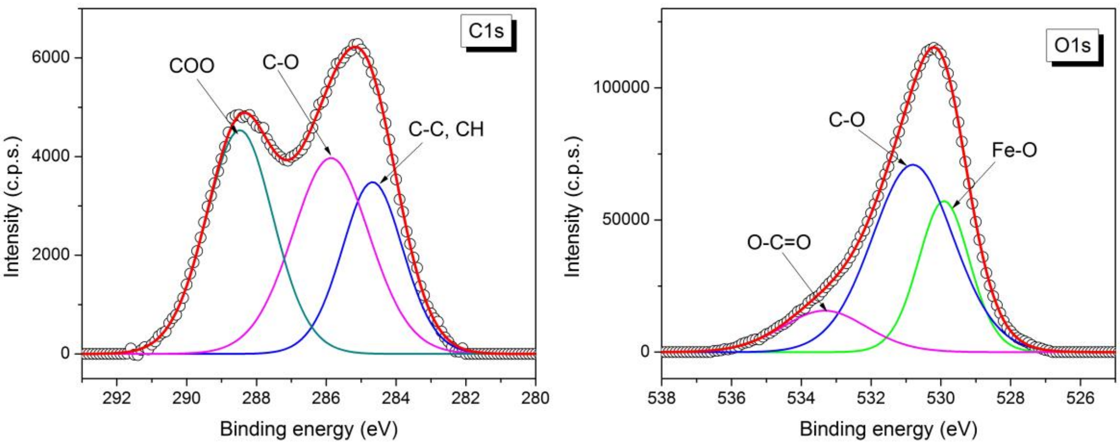

Figure S10. XPS spectra of **3g**-3 h.

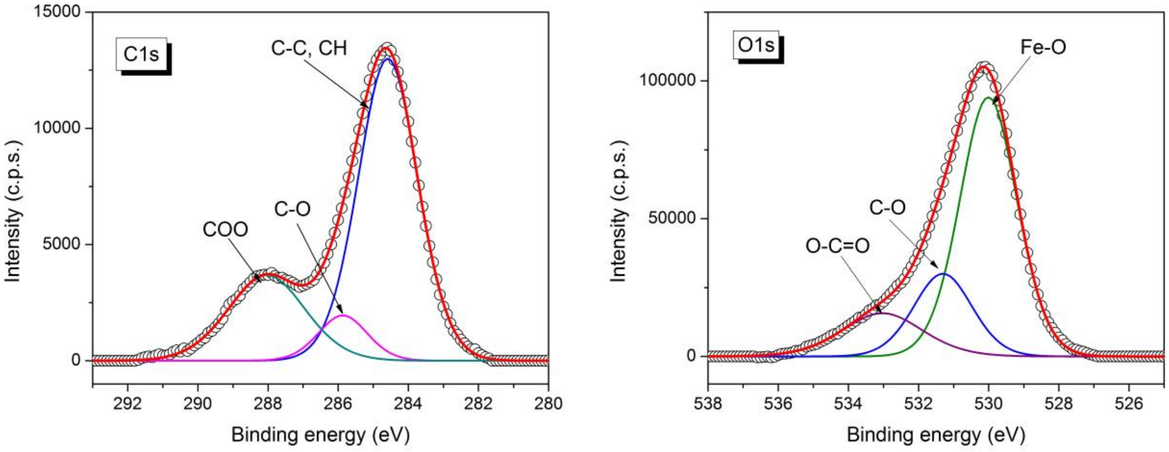

Figure S11. XPS spectra of **3h**-3 h.

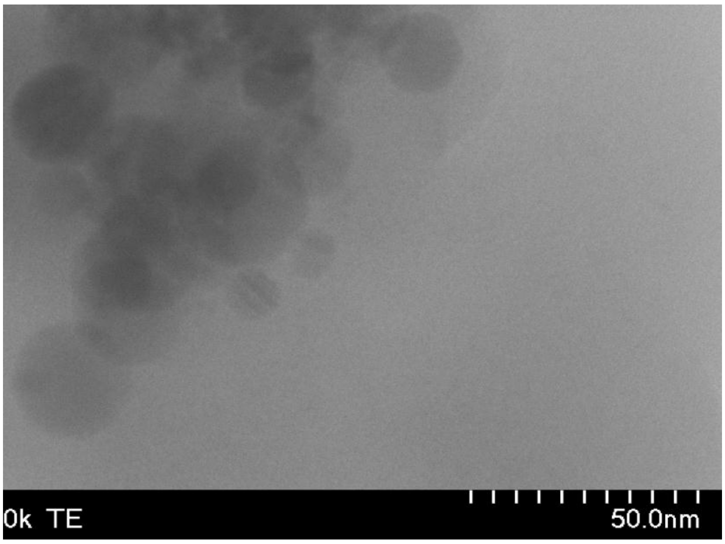

Figure S12. TEM image of **3c**-3 h (Fe_3O_4 covered with phospho-L-ascorbic acid).

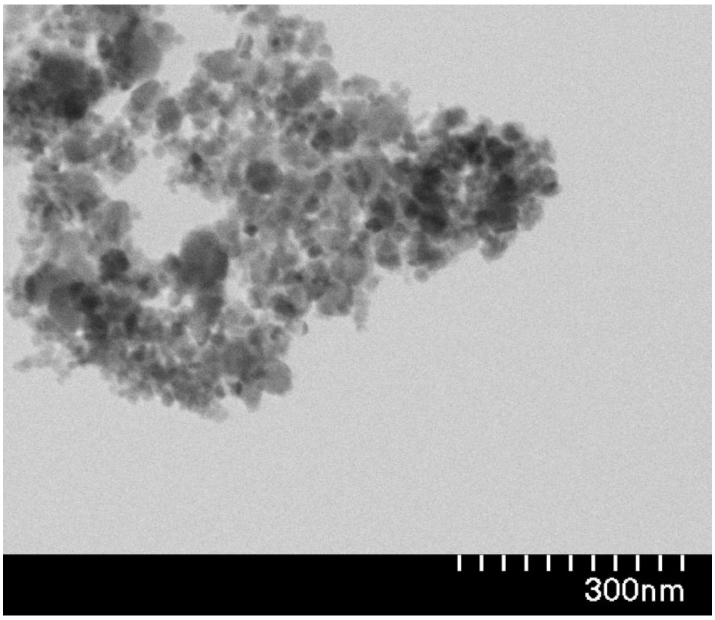

Figure S13. TEM image of **3e**-24 h (Fe_3O_4 covered with glycolic acid).

© 2016 by the authors. Submitted for possible open access publication under the terms and conditions of the Creative Commons Attribution (CC-BY) license (http://creativecommons.org/licenses/by/4.0/).
